# Intercalated theophylline-smectite hybrid for pH-mediated delivery

**DOI:** 10.1007/s13346-018-0478-8

**Published:** 2018-01-23

**Authors:** Vivek Trivedi, Uttom Nandi, Mohammed Maniruzzaman, Nichola J. Coleman

**Affiliations:** 1 Faculty of Engineering and Science, University of Greenwich, Chatham Maritime, Chatham, Kent, ME4 4TB UK; 2 Pharmaceutics Research Laboratory, School of Life Sciences, University of Sussex, Falmer, UK

**Keywords:** Smectite, Montmorillonite, Theophylline, Intercalation, Drug release

## Abstract

On the basis of their large specific surface areas, high adsorption and cation exchange capacities, swelling potential and low toxicity, natural smectite clays are attractive substrates for the gastric protection of neutral and cationic drugs. Theophylline is an amphoteric xanthine derivative that is widely used as a bronchodilator in the treatment of asthma and chronic obstructive pulmonary disease. This study considers the in vitro uptake and release characteristics of the binary theophylline-smectite system. The cationic form of theophylline was readily ion exchanged into smectite clay at pH 1.2 with a maximum uptake of 67 ± 2 mg g^−1^. Characterisation of the drug-clay hybrid system by powder X-ray diffraction analysis, Fourier transform infrared spectroscopy, differential scanning calorimetry and scanning electron microscopy confirmed that the theophylline had been exclusively intercalated into the clay system in an amorphous form. The drug remained bound within the clay under simulated gastric conditions at pH 1.2; and the prolonged release of approximately 40% of the drug was observed in simulated intestinal fluid at pH 6.8 and 7.4 within a 2-h timeframe. The incomplete reversibility of the intercalation process was attributed to chemisorption of the drug within the clay lattice. These findings indicate that smectite clay is a potentially suitable vehicle for the safe passage of theophylline into the duodenum. Protection from absorption in the stomach and subsequent prolonged release in the small intestine are advantageous in reducing fluctuations in serum concentration which may impact therapeutic effect and toxicity.

## Introduction

Aluminosilicate clays are widely used in the pharmaceutical industry as stabilising and suspending agents, rheology modifiers and texture enhancers in various dosage forms [1, 2]. Smectites (a.k.a. bentonites) are a family of 2:1 clays whose principal repeating layer system comprises an octahedral magnesium or aluminium oxide sheet bound between two tetrahedral silicate sheets [1]. In montmorillonite, (Na,Ca)_0.33_(Al,Mg)_2_(Si_4_O_10_)(OH)_2_·nH_2_O, two thirds of the octahedral sites are occupied by Al^3+^ ions, and in saponite, Ca_0.25_(Mg,Fe)_3_((Si,Al)_4_O_10_)(OH)_2_·n(H_2_O), the octahedral sites are fully occupied by Mg^2+^ ions. Lattice substitution of Mg^2+^ for Al^3+^ in montmorillonite, and Al^3+^ for Si^4+^ substitution in saponite, confers a negative charge on the layers which is balanced by labile interlayer Na^+^ and Ca^2+^ cations. In addition to the charge-balancing cations, water molecules are also present between the layers, both of which are readily exchangeable.

Montmorillonite, saponite and their mixtures are popular options in clay-drug hybrid controlled release systems for oral, transdermal and topical administration [1–5]. Cationic drugs are particularly suitable for intercalation within these clays, as they are conveniently exchanged for the interlayer cations and their subsequent electrostatic interaction with the negatively charged layers extends their release. In addition to modified release, intercalation within the clay can also afford protection from selected in vivo environments such as gastric acidity.

**Fig. 1:**
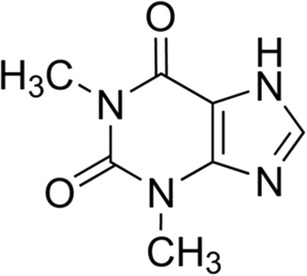
The structure of theophylline

Theophylline, C_7_H_8_N_4_O_2_, is a xanthine derivative (shown in Fig. 1) which acts as an adenosine receptor antagonist and phosphodiesterase inhibitor [6]. It is used as a bronchodilator in the treatment of asthma and chronic obstructive pulmonary disease. Common adverse effects include nausea, vomiting, diarrhoea, hypokalaemia, rhabdomyolysis, ventricular arrhythmia, tachycardia and seizures [7]. Theophylline is 90–100% bioavailable. Its therapeutic serum concentration range is 5–20 mg dm^−3^ and it possesses a relatively short half-life of approximately 6 h [6, 8]. Accurate prediction of the required dose is complicated by large interpatient variability in absorption and elimination rates [6, 8]. For example, elevated gastric acidity is known to enhance the absorption of theophylline in the stomach which is associated with increased side effects and high fluctuations in serum concentration [8].

Recent studies have indicated that the incorporation of smectite clays in multicomponent theophylline formulations can provide sustained release which affords the potential to reduce both dosing frequency and adverse effects [3, 9]. The principal objective of the present study was to determine whether the intercalation of theophylline in smectite clay is able to protect the drug from the acidic gastric environment and to enable its controlled release in the small intestine. The uptake kinetics of theophylline by a commercial pharmaceutical grade smectite clay (VEEGUM®, Vanderbilt Minerals, LLC, CT, USA) were monitored as a function of theophylline concentration. Equilibrium uptake isotherms were obtained as functions of both theophylline and smectite concentrations. The nature of the drug-clay interactions were determined by powder X-ray diffraction analysis, Fourier transform infrared spectroscopy, differential scanning calorimetry and scanning electron microscopy. The release profiles of theophylline from the drug-clay hybrid were obtained in phosphate-buffered saline at pH 7.4, simulated gastric fluid at pH 1.2 and simulated intestinal fluid at pH 6.8 and 7.4.

## Materials and methods

### Materials and characterisation

The smectite clay (SM) used in this study was supplied by Vanderbilt Minerals, LLC (Norwalk, CT, USA) and is commercially available as VEEGUM® F. VEEGUM® F is a pharmaceutical grade mixture of natural montmorillonite and saponite clays that has been ‘micronized’ to a mean particle size of 100 μm [10]. The smectite was heated at 100 °C in air prior to use to remove adsorbed water. Theophylline (TP) was purchased from BASF (Ludwigshafen am Rhein, Germany) in the form of a white anhydrous powder with a purity of > 99%. All other analytical grade reagents were obtained from Fisher Scientific (Loughborough, UK) and used as received.

The crystalline structures of the smectite clay and theophylline drug were confirmed by powder X-ray diffraction analysis (XRD) using a Bruker D8 ADVANCE diffractometer with Cu Kα  = 1.5406 Å at a step size of 0.02° in the 2θ range from 2 to 70° and a measuring time of 140 s per step. Fourier transform infrared (FTIR) spectra of the clay and drug were acquired using a Perkin Elmer Spectrum Two spectrometer between 450 and 4000 cm^−1^ wavenumbers, with 10 scans at a resolution of 4 cm^−1^. Secondary electron images were obtained from uncoated samples attached to carbon tabs on an Hitachi SU8030 scanning electron microscope with an accelerating voltage of 0.7 kV. Differential scanning calorimetry was carried out on the clay and drug using a Mettler-Toledo DSC823e calorimeter. Thermograms were collected on 2–3 mg of material in sealed, pierced aluminium pans between 25 and 300 °C at a heating rate of 10 °C min^−1^ under nitrogen.

### Theophylline uptake

The initial rates of uptake of theophylline by the smectite clay were determined by batch sorption at 50 °C as functions of theophylline concentration. In each case, 1.25 g of smectite were dispersed in 25 cm^3^ of 0.1 N HCl_(aq)_ at pH 1.2 by sonication for 30 min to enable the clay to swell. 25, 50, 75, 125 or 175 mg of theophylline were then added to the suspension under stirring at 250 rpm (to produce samples labelled; TP20SM, TP40SM, TP60SM, TP100SM and TP140SM, respectively, where the number indicates the TP/SM ratio in mg g^−1^). Contact times for specimens were at 30-s intervals for 5 min, after which, the supernatant liquors were recovered by centrifugation at 7500 rpm for 2 min and subsequent filtration with a 0.45-μm syringe filter. The concentration of theophylline remaining in solution was then determined by UV-vis spectroscopy at 270 nm using an Hitachi U-2900 spectrophotometer [2]. Each experiment was carried out in triplicate and the uptake of theophylline by smectite was calculated by dividing the difference between the initial and final masses of theophylline in solution (in mg) by the mass of smectite present (in g).

Isotherm analysis of the uptake of theophylline by smectite as a function of theophylline concentration was carried out by batch sorption at 50 °C. The procedure described above was repeated with a contact time of 60 min for each sample type to obtain equilibrium uptake data which were then plotted against initial theophylline concentration. Additionally, in each case, the theophylline-loaded clay particles were recovered, washed five times with 0.1 N HCl_(aq)_ and dried at 60 °C in air to constant mass. The recovered clay-drug hybrid materials were stored in air-tight polypropylene containers at room temperature for characterisation by XRD and DSC analyses (as outlined previously). A dried sample of the swollen smectite (SM-H_2_O) following immersion in 0.1 N HCl_(aq)_ at pH 1.2 for 30 min was also analysed by XRD, FTIR and DSC for comparison.

Isotherm analysis of theophylline uptake as a function of smectite concentration was also carried out at 50 °C under the same experimental conditions described previously. Thus, isotherm data were collected from batch sorption experiments of varying smectite concentration (5, 10, 20, 30, 40 and 50 mg cm^−3^) at a constant initial theophylline concentration of 3 mg g^−1^ with a contact time of 60 min. Again, in each case, the equilibrium extent of theophylline uptake by smectite was calculated via a simple mass balance for theophylline.

### In vitro release of theophylline

The theophylline-smectite hybrid (viz. TP60SM) selected for the in vitro release study was prepared by the batch sorption method described above using 50 mg cm^−3^ of smectite suspended in 0.1 N HCl_(aq)_ containing 3 mg cm^−3^ of theophylline at 50 °C for 60 min. The clay-drug hybrid was recovered by centrifugation, washed five times with 0.1 N HCl_(aq)_ and dried at 60 °C in air to constant mass prior to characterisation by XRD, FTIR, SEM and DSC and in vitro release analysis.

In vitro release of theophylline from the drug-clay hybrid was monitored in phosphate-buffered saline (PBS) at pH 7.4 [11], simulated gastric fluid (SGF) at pH 1.2 [12] and simulated intestinal fluid (SIF) at pH 6.8 and 7.4 [13]. Theophylline release was also observed in modified SIF dissolution media to which were added either 8.0 mg cm^−3^ NaCl (i.e. the quantity present in PBS) or 0.4 mg cm^−3^ NaCl (i.e. a smaller incremental quantity), viz. SIF-8.0 and SIF-0.4, respectively, to investigate the influence of ionic concentration on the release behaviour. The principal compositions of the dissolution media used in this study are listed in Table 1, and it should be noted that the final pH values were achieved by additions of either 0.2 M HCl_(aq)_ or 0.1 M NaCl_(aq)_.

**Table 1:** Compositions of dissolution media per cubic decimetre [11–13]

Dissolution medium	PBS	SGF	SIF	SIF-8.0	SIF-0.4
NaCl (g)	8.0	–	–	8.0	0.4
KCl (g)	0.2	3.73	–	–	–
KH_2_PO_4_ (g)	0.24	–	6.85	6.85	6.85
Na_2_HPO_4_ (g)	1.44	–	–	–	–
NaOH (g)	–	–	0.896	0.896	0.896
0.2 M HCl_(aq)_ (cm^3^)	–	425	–	–	–

Theophylline release profiles were obtained by suspending 200 mg of the drug-clay hybrid in 250 cm^3^ of the selected dissolution medium under continuous stirring at 100 rpm. Three cubic centimetre aliquots of the supernatant were withdrawn, filtered through 0.45-μm syringe filters and analysed by UV-vis spectroscopy at intervals up to 180 min. At each interval, the withdrawn aliquot was replaced by 3 cm^3^ of fresh medium, and the appropriate correction was applied to the calculation of the percentage of theophylline released from the hybrid. Each experiment was carried out in triplicate.

## Results

### Characterisation of smectite

**Fig. 2:**
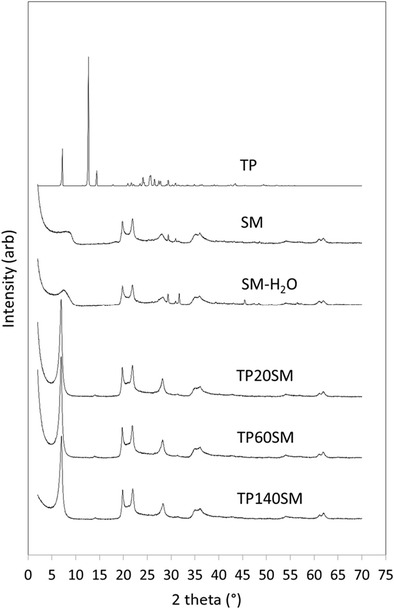
Powder XRD patterns of theophylline, smectite and clay-drug hybrids

The powder XRD patterns of randomly orientated samples of the commercial pharmaceutical grade smectite clay used in this study after drying at 100 °C (SM) and after swelling in water (SM-H_2_O) are shown in Fig. 2. These XRD patterns are consistent with those in the literature for other smectite mixtures comprising montmorillonite and saponite [14–16]. The basal spacing of anhydrous smectite is typically 10 Å which increases incrementally to 12.5, 15.5 and 18.5 Å with the incorporation of 1, 2 and 3 homogeneous water layers between each of the aluminosilicate sheets [15]. The basal spacings of SM and SM-H_2_O are ~ 11 and 12.2 Å, respectively, indicating that the dried, as received, clay is not entirely anhydrous and that the water-swollen clay used for the uptake of theophylline possesses less than one complete water layer. The very broad basal reflections observed for both SM and SM-H_2_O are indicative of poor organisation of the layers in the c-axis direction.

**Fig. 3:**
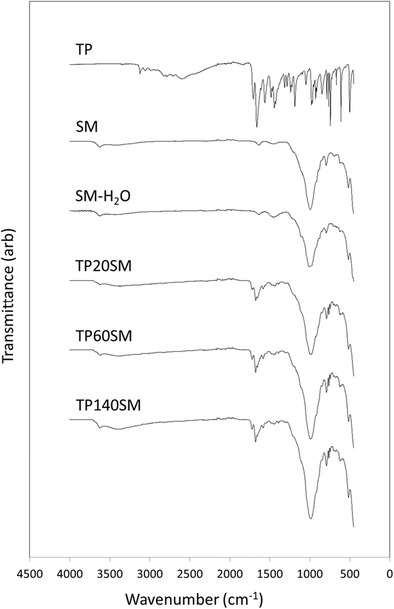
FTIR spectra of theophylline, smectite and clay-drug hybrids

The FTIR spectra of SM and SM-H_2_O, shown in Fig. 3, typify those of smectite clays [16, 17]. Stretching modes of structural hydroxyl groups are assigned to the discrete signal at 3615 cm^−1^ and those of adsorbed and bound water appear as a very broad signal centred around 3440 cm^−1^ [16, 17]. Bending modes of water occur at 1625 cm^−1^, and the signal at 1445 cm^−1^ is attributed to trace quantities of calcium carbonate. Various Si-O-Si lattice vibrations give rise to the bands at 990, 800 and 690 cm^−1^, and the signal at 525 cm^−1^ is assigned to Si-O-Al modes.

**Fig. 4:**
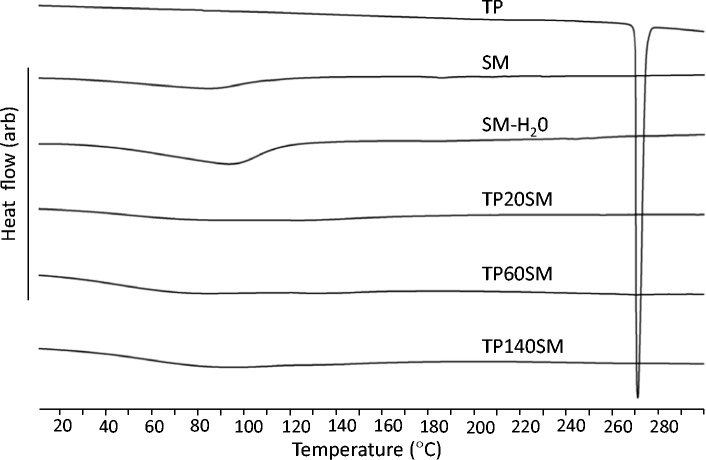
DSC curves of theophylline, smectite and clay-drug hybrids

As anticipated, the DSC thermograms of SM and SM-H_2_O, presented in Fig. 4, are essentially uneventful with the exception of the endothermic removal of adsorbed water between 40 and 130 °C. Understandably, the endotherm is greater in the case of the more hydrated clay, SM-H_2_O.

**Fig. 5:**
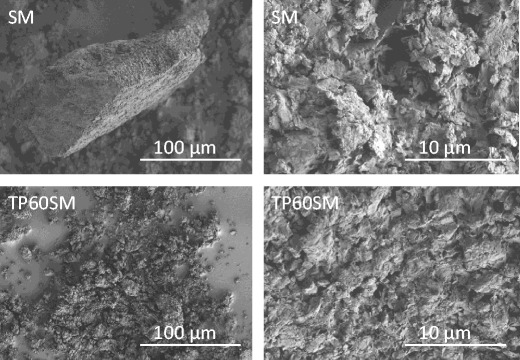
Secondary electron images of smectite and clay-drug hybrid

Secondary electron images of SM (Fig. 5) show that this material is highly polydispersed with granules of varying aspect ratio and maximum particle dimension of approximately 100 μm. Higher magnification also reveals the platy texture of the clay particles within the granules.

### Characterisation of theophylline

The powder XRD pattern of theophylline (TP) shown in Fig. 2 and the sharp melting point at 271 °C in the corresponding DSC curve (Fig. 4) both confirm that the material used in this study is pure crystalline anhydrous theophylline [18, 19]. The FTIR spectrum of theophylline (Fig. 3) also closely resembles those in the scientific literature [19, 20]. Various bands in the region 3440–2460 cm^−1^ are assigned to the stretching modes of the N-H group and to the aliphatic and aromatic C-H bonds present in theophylline. Characteristic stretching of the carbonyl groups occurs at 1710 and 1665 cm^−1^ and the amine N-H stretching gives rise to the signal at 1565 cm^−1^.

### Uptake of theophylline by smectite

**Fig. 6:**
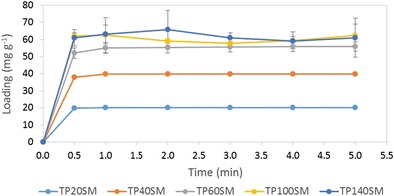
Uptake profiles of theophylline by smectite as functions of theophylline concentration

The rates of uptake of theophylline by the smectite clay at pH 1.2 as functions of theophylline concentration are plotted in Fig. 6. The initial rate of uptake is seen to increase with theophylline concentration, and in all cases, equilibrium is achieved within 1 min. Below pH 4, the imine nitrogen atom of theophylline becomes protonated and the resulting cation is stabilised by the electron resonance of the five-membered aromatic ring and inductive effects [21]. The rapid uptake of theophylline by smectite at pH 1.2 is indicative of the favourable electrostatic interaction between the protonated cationic form of the drug and the negatively charged clay sheets. It should be noted that the mechanism of interaction between smectite clays and theophylline is reported to proceed via a two-step process which involves initial rapid cation exchange followed by chemisorption [22].

**Fig. 7:**
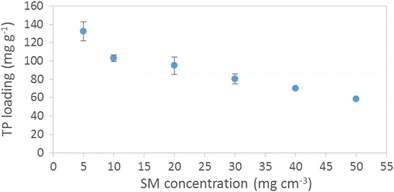
Equilibrium uptake of theophylline as a function of smectite concentration

**Fig. 8:**
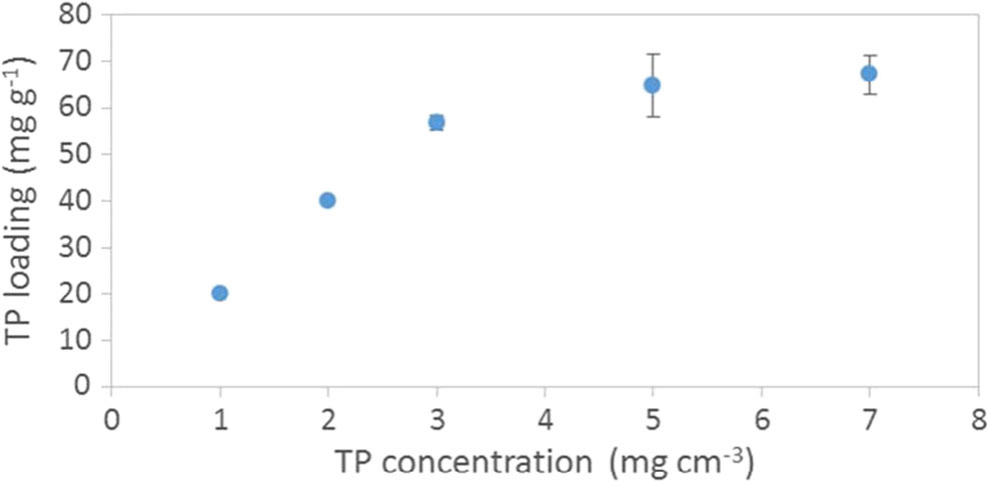
Equilibrium uptake of theophylline as a function of theophylline concentration

The equilibrium uptake of theophylline as a function of smectite concentration is shown in Fig. 7. The quantity of adsorbed drug per unit mass of smectite decreases non-linearly with increasing smectite concentration which indicates that the clay presents multiple adsorption sites of differing energy. The concentration of smectite that gives the lowest drug-clay loading per unit mass of clay (i.e. 50 mg cm^−3^) was selected for further isotherm analysis. The rationale for this choice is that this system possesses the most energetically uniform drug-clay interactions. Under the selected batch conditions, a Langmuir-type isotherm is obtained for the equilibrium uptake of theophylline as a function of drug concentration (Fig. 8) and demonstrates that the maximum loading capacity for this drug-clay system is 67 ± 2 mg g^−1^.

### Characterisation of theophylline-smectite hybrid

Samples corresponding to alternate points on the isotherm plotted in Fig. 8 were characterised by XRD, FTIR and DSC to determine the nature of the drug-clay interaction. Drug-clay hybrids, TP20SM, TP60SM and TP140SM, were prepared by contacting 50 mg cm^−3^ smectite with 1, 3 or 7 mg cm^−3^ solutions of theophylline, respectively, for 60 min.

Powder XRD patterns of TP20SM, TP60SM and TP140SM are shown in Fig. 2. The basal spacing of the smectite is seen to increase to 14.3 Å for all hybrid samples, demonstrating that the drug is intercalated within the clay. The sharp principal reflections for crystalline theophylline (at 2θ = 7.21, 12.69 and 14.36°) are absent from these XRD traces, and a weak broad signal at 13.59° arises from the intercalation of the drug. The comparatively sharp basal reflections of the drug-intercalated smectite samples indicate a superior stacking order along the c-axis relative to that of the original clay. DSC analysis confirms the absence of a theophylline melting event within the hybrid systems demonstrating that the intercalated drug is present in an amorphous form (Fig. 4). Secondary electron images of sample TP140SM are shown in Fig. 5 and illustrate that the larger granules tend to disintegrate during the drug intercalation process. However, the platy morphology of the clay appears unaffected by intercalation and there is no evidence for the precipitation of theophylline on the surface of the clay platelets.

Characteristic stretching vibrations of the carbonyl groups of theophylline are present in the FTIR spectra of the drug-clay hybrids (Fig. 3) at 1710 and 1665 cm^−1^ which are partially obscured by the bending modes of water in the clay. A shift in the position of the amine stretching signal of the hybrids relative to that of crystalline theophylline is observed from 1565 to 1578 cm^−1^. This may be attributed to the electrostatic interaction between the drug and clay; although, this cannot be fully confirmed as shifts may also arise from the protonation of the drug and its phase change from the crystalline to the amorphous state.

### In vitro release of theophylline from the drug-clay hybrid

The in vitro release behaviour of theophylline from drug-clay hybrid TP60SM was monitored in PBS, SGF and SIF at pH 6.8 and pH 7.4 (which represent the environments of the duodenum and ileum, respectively). This hybrid has a drug loading of 57 ± 2 mg g^−1^ and was selected for the release study as it represents the most energetically uniform adsorption system with a loading below the theoretical maximum value (to prevent any ‘initial burst’ of loosely adsorbed drug).

**Fig. 9:**
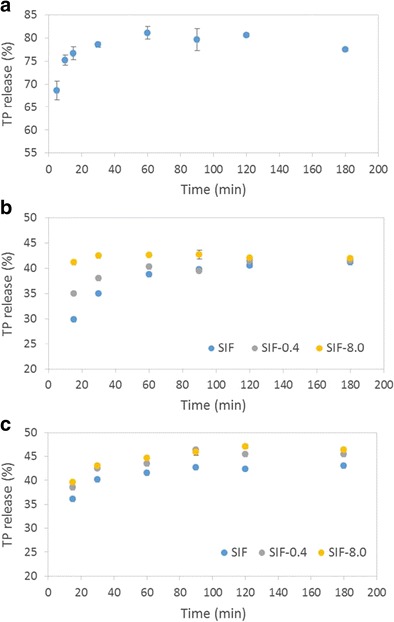
Release profiles of theophylline in **a** PBS, **b** SIF at pH 6.8 and **c** SIF at pH 7.4

Under the selected experimental conditions, theophylline is steadily released in PBS at pH 7.4 to a maximum extent of 80% after 60 min (Fig. 9a). The release profile does not conform to a simple diffusion model which indicates that the complex nature of interactions between the drug and clay lattice dictate the release behaviour [3, 22].

No detectible release of theophylline was observed from the drug-clay hybrid in simulated gastric fluid at pH 1.2 during an extended 72-h observation period. This finding demonstrates that the intercalation of theophylline in smectite at pH 1.2 is not readily reversible at the same pH despite the presence of potentially exchangeable K^+^ cations in the supernatant liquor.

The release profiles of theophylline in simulated intestinal fluid at pH 6.8 and at pH 7.4 are plotted in Fig. 9b, c, respectively. These data show that theophylline is released more rapidly at the higher pH with a maximum release of 43% after 60 min. Incremental dissolution of the drug at pH 6.8 continued throughout the 3-h observation period to give a maximum release of 41%.

Since the maximum release of theophylline in SIF is observed to be approximately 50% lower than that in PBS, the composition of SIF was modified to match the Na^+^ ion concentration of PBS (by addition of 8.0 mg cm^−3^ of NaCl). SIF was also prepared with the addition of a relatively low level (0.4 mg cm^−3^) of Na^+^ ions to determine the influence of concentration of this potentially exchangeable cation on the release of the drug. Accordingly, the release of theophylline in SIF-8.0 and SIF-0.4 at pH 7.4 and pH 6.8 is plotted in Fig. 9b, c, respectively. In both cases, the rate of release was enhanced as the Na^+^ ion concentration increased, with this effect being more pronounced at pH 6.8. It is clear that the mass action of cations in the supernatant solution accelerates the release of the drug, but has little impact on the ultimate quantity of theophylline that is discharged from the clay. Again, none of the release profiles obtained in the various SIF media conformed to a simple diffusion model [3, 22].

At present, the reason for the superior release of theophylline in PBS compared with that in the original and modified SIF media is unknown, but presumably relates to the influence of different concentrations of anionic phosphate species present in these liquors (Table 1).

## Discussion

The large specific surface area, high adsorption and cation exchange capacities, swelling potential and low toxicity of smectites have been exploited in a wide range of formulations for modulating drug delivery [1–5, 9, 16]. They are particularly suitable for the gastric protection of cationic drugs and are also under consideration as gastrointestinal decontamination agents to mitigate the absorption of ingested toxins [23].

The protonated cationic forms of basic and amphoteric ionisable drugs (p*K*a > 7) are readily intercalated between the negatively charged sheets of smectite clays under aqueous acidic conditions [23, 24]. At low pH, such as that encountered in the stomach, the cationic form of the drug persists and the strong electrostatic drug-clay interactions continue to bind the drug to the clay matrix preventing its release. In addition, subsequent to the initial intercalation by ion exchange, the drug may become chemisorbed to the clay which will ultimately limit the maximum extent to which the drug can be released [22].

A number of recent studies reports the release of theophylline from multicomponent formulations containing smectite clays among numerous other constituents such as polymers, hydrogels and other common excipients [2, 3, 9]. However, there is a surprising paucity of information on the binary theophylline-smectite system in the current literature [22, 23].

The present study demonstrates that the maximum uptake of theophylline by the selected commercial pharmaceutical grade smectite at pH 1.2 was 67 ± 2 mg g^−1^. This is consistent with the findings of Mináriková et al. [23] who report the same maximum loading of theophylline in a different commercial smectite clay under simulated gastrointestinal conditions.

The present study also confirms that the adsorbed theophylline was exclusively intercalated into the clay lattice in an amorphous form which was not released under simulated gastric conditions (at pH 1.2). As previously mentioned, enhanced absorption of theophylline from the stomach is associated with increased side effects and high fluctuations in serum concentration, and in this respect, smectite clay affords a potentially suitable vehicle for the passage of the drug into the small intestine. Indeed, approximately 40% of the intercalated theophylline was steadily released into simulated intestinal fluid at pH 6.8 and 7.4 within a 2-h timeframe. The observed release profiles did not conform to a diffusion model indicating that the complex interactions between the drug and clay dictate the release kinetics.

The reported impact of smectite clay on the release behaviour of theophylline from multicomponent formulations is highly variable [2, 3, 9]; although, in general, prolonged release is observed when the drug is incorporated under neutral or acidic aqueous conditions. Dry-blended tableted formulations do not exploit the modulating potential of smectite, since, under these processing conditions, theophylline is not able to interact with the clay [2].

## Conclusions

The cationic form of theophylline was readily ion exchanged into smectite clay under aqueous acidic conditions (pH 1.2) at 50 °C with a maximum uptake of 67 ± 2 mg g^−1^. Characterisation of the drug-clay hybrid by powder X-ray diffraction analysis, Fourier transform infrared spectroscopy, differential scanning calorimetry and scanning electron microscopy confirmed that the theophylline had been intercalated into the clay in an amorphous form. The drug remained bound within the clay under simulated gastric conditions (pH 1.2), and the retarded release of approximately 40% of the drug was observed in simulated intestinal fluid at pH 6.8 and 7.4 within a 2-h timeframe. The incomplete reversibility of the intercalation process was attributed to chemisorption of the drug within the clay lattice. These findings indicate that smectite clay is a potentially suitable platform for the safe passage of theophylline into the duodenum. Protection from absorption in the stomach and subsequent prolonged release in the small intestine are advantageous with respect to detrimental fluctuations in serum concentration.
